# Evaluation of a therapeutic vaccine for the prevention of recurrent urinary tract infections versus prophylactic treatment with antibiotics

**DOI:** 10.1007/s00192-012-1853-5

**Published:** 2012-07-18

**Authors:** M. F. Lorenzo-Gómez, B. Padilla-Fernández, F. J. García-Criado, J. A. Mirón-Canelo, A. Gil-Vicente, A. Nieto-Huertos, J. M. Silva-Abuin

**Affiliations:** 1 Servicio de Urología, Complejo Asistencial Universitario de Salamanca, Paseo San Vicente 58-182, Instituto de Investigación Biomédica de Salamanca, 37007 Salamanca, Spain; 2 Departamento de Cirugía, Universidad de Salamanca, Salamanca, Spain; 3 Departamento de Medicina Preventiva y Salud Pública, Universidad de Salamanca, Salamanca, Spain; 4 Medicina de Familia y Comunitaria del Complejo Asistencial de Zamora, Universidad de Salamanca, Salamanca, Spain

**Keywords:** Recurrent urinary tract infections, Bacterial vaccine

## Abstract

**Introduction and hypothesis:**

Urinary tract infections (UTIs) are considered the most common bacterial infections, especially in women. The objective of this study was to evaluate the use of the sublingual bacterial vaccine Uromune® in order to prevent recurrent UTIs (RUTIs).

**Methods:**

This study was conceived as a multicenter observational study. The clinical history of 319 women who presented at least 2 episodes of UTI in the last 6 months or 3 in 12 months was reviewed. Data related to treatment and clinical evolution were recorded and analyzed. A total of 159 patients received prophylactic treatment with Uromune® for a period of 3 months (group A) and 160 with sulfamethoxazole/trimethoprim 200/40 mg/day for a period of 6 months (group B). Uromune® contained an inactivated bacterial cell suspension of selected strains of *Escherichia coli*, *Klebsiella pneumoniae*, *Proteus vulgaris*, and *Enterococcus faecalis*.

**Results:**

Patients in group A experienced a highly significant reduction in the number of infections compared to patients in group B. In the first 3 months, the mean number of infections was 0.36 versus 1.60 (*P* < 0.0001), respectively. A significant reduction was also observed after 9 and 15 months (*P* < 0.0001). The numbers of patients who did not have any UTI at 3, 9, and 15 months were 101, 90, and 55 in group A versus 9, 4, and 0 in group B (*P* < 0.0001).

**Conclusions:**

The results obtained in this study favor the use of this bacterial-based therapeutic vaccine as an effective strategy to reduce frequency, duration, severity, and costs of RUTIs.

## Introduction

Urinary tract infections (UTIs) are considered the most common bacterial infections [1, 2], especially in women. The bladder is the most prevalent site of infection (cystitis). Some authors stated that women are 30 times more prone to have UTIs than men [3], while other authors reported a 8:1 ratio [4]. Because 50–60 % of women report at least one UTI in their lifetime, UTIs have become a common condition diagnosed and treated by urologists, gynecologists, and other health care providers [1].

At least one fourth of these patients will have a recurrence within a year [2], and 22 % will have recurrent urinary tract infections (RUTIs). Infections are considered recurrent when patients have three or more culture-documented infections in a year or two or more in 6 months [5].

The European Association of Urology defines a significant bacteriuria as ≥ 10^3^ uropathogens/ml of midstream urine in acute uncomplicated cystitis in women, ≥ 10^4^ uropathogens/ml of midstream urine in acute uncomplicated pyelonephritis in women, ≥ 10^5^ uropathogens/ml of midstream urine in women, or ≥ 10^4^ uropathogens/ml of midstream urine in men (or in straight catheter urine in women) with complicated UTI (in a suprapubic bladder puncture specimen, any count of bacteria is relevant) [6].

On average, each episode of acute UTI in premenopausal women is associated with 6.1 days of symptoms, 2.4 days of school or work absenteeism, and 0.4 days in bed [1]. Besides the clinical impact on the health and quality of life, RUTIs have a great economic impact. An annual cost of US$2.5 billion is estimated in the USA [5]. Bacteriuria is found in 2–3 % of women 15–24 years of age, 20 % in women 65–80 years of age, and 25–50 % in women greater than 80 years of age [7]. The population of women greater than 65 years of age in the USA is projected to double between 2000 and 2030 [8]. Therefore, it could be anticipated that UTIs will increase in the upcoming years. Aside from being common in the community, 2 % of the hospitalized patients acquire UTIs. In the 1980s, nosocomial infections accounted for more than 500,000 per year [9, 10].

Continuous prophylaxis with antibiotics is the recommended initial therapy for RUTIs. Sulfamethoxazole/trimethoprim (SMX/TMP) for a period of 6 months is the most commonly recommended option [11]. However, the continuous use of antibiotics is not free of risk. Multiresistance of the bacteria to antibiotics is widely increasing, which creates the dramatic situation that more than 40 % of the bacterial strains are resistant to available antibiotics in some regions of the world. An added problem is the high incidence of adverse reactions associated with the use of antibiotics and other chemotherapeutics.

Therefore, it is reasonable to consider other preventive strategies such as those that reinforce the natural mechanisms of pathogen defense.

The objective of this study was to evaluate clinical benefit obtained with the prophylactic use of a bacterial-based sublingual vaccine (Uromune®) compared with prophylactic treatment with SMX/TMP to prevent recurrences of UTIs.

## Materials and methods

### Study design

This study was conceived as a multicenter retrospective observational study. It was approved by the Ethics Committee of the University Hospital of Salamanca (Spain) and was accepted by the Spanish Health Authorities (*Agencia Española del Medicamento*).

### Patient population

The sample size of the number of clinical histories to review was calculated based on the data of Palou et al. [12] describing that more than 11.8 of the population have more than 2 UTIs. Expecting to have a 65 % reduction in the number of UTIs, for an α = 0.05 and a power of 80 % (error protection of 7.84) [13], the number of clinical histories to review was a minimum of 314.

Finally, the clinical history of 319 women who presented with at least 2 episodes of UTI in the last 6 months or 3 in the last 12 months was reviewed. Data related to medical and surgical background, gynecological/obstetric records, sexual habits, usual treatment, specific treatment, and evolution were recorded and analyzed. Between groups there were no clinically relevant differences regarding age, hormonal status, coital activity, or usual treatment. The ethnic group and the dietetic habits were the same in all patients. Epidemiological data on age, months of evolution of RUTI, and the mean number of UTIs and positive urocultures (UC+) in a period of 6 months are shown in Table 1. Once the clinical histories were reviewed, 159 patients were treated with Uromune® (group A) and 160 with SMX/TMP (group B). In this review, we found that the clinical history of the patients who suffered an UTI had the corresponding UC and were treated with different antibiotics (ciprofloxacin, cefuroxime, amoxicillin/clavulanic acid, etc.).

**Table 1:** Demographic data of the patients evaluated

	Group A	Group B	*P*
Age	47.7 (45.0–50.4)	48.1 (45.1–51.0)	0.8536
Age range	16-85	16-87	
Time of evolution (months)	56.7 (44.7–68.7)	59.2 (47.8–70.6)	0.7641
Mean number of UTI/6 months^a^	3.2 (2.7–3.7)	3.1 (2.8–3.3)	0.2789
Mean number of UC+/6 months^a^	2.4 (2.0–2.8)	2.2 (2.0–2.4)	0.6392

^a^Mean number of episodes in a 6-month period

### Treatments

Uromune® is a commercially available bacterial vaccine produced (in Spain as a named patient preparation) by Inmunotek (Madrid, Spain) and marketed by Q-Pharma (Alicante, Spain). The vaccine consisted of two vials containing a suspension of 10^9^ inactivated whole bacteria/ml. The vaccine is a mixture of equal amounts of selected strains of *Escherichia coli*, *Klebsiella pneumoniae*, *Proteus vulgaris*, and *Enterococcus faecalis*. These microorganisms are considered to produce the majority of RUTIs in Spain [14]. The delivery route was through the sublingual mucosa and the dose was 2 puffs of 100 μl each (10^8^ bacteria/puff) daily avoiding the concomitant intake of food or beverage. The delivered dose was maintained under the tongue for a period of 1–2 min and then swallowed. The patients in group A received this treatment during a period of 3 months.

SMX/TMP (200/40 mg/day) was administered orally to patients in group B as a prophylactic treatment for a period of 6 months [11, 15, 16].

The clinical history of all patients contained the data concerning each episode of UTI. Furthermore, group A had a routine control after completion of the 3-month treatment with Uromune® and group B after 6 months of SMX/TMP.

### Evaluation

The following data were collected before the initiation of the corresponding treatment and after 3, 9, and 15 months: (1) number of UTIs, (2) time of evolution of RUTIs before initiation of treatment, and (3) number of UC+.

### Statistics

The Excel spreadsheet (Microsoft Corp., Redmond, WA, USA) and the statistical software SPSS v.11.0 (SPSS Inc., Chicago, IL, USA) were used. Descriptive statistics of the number of UTIs and UC+ were expressed as the mean in the corresponding period with 95 % confidence intervals (CI). Student’s *t* test was used to compare the number of UTIs and UC+ between both groups before the initiation of treatment and 3, 9, and 15 months of evolution. Repeated measures analysis of variance (ANOVA) was used to evaluate the evolution of the number of UTIs and UC+ of each group at 3, 9, and 15 months of observation. Fisher’s exact test was used to compare between both groups the number of patients who did not experience any UTIs or UC+ after 3, 9, and 15 months.

The relationship between the mean number or UTIs or UC+ and the time of observation (3, 9, and 15 months) was estimated by the regression line analysis Y = a + bX, in which *Y* is the number of UTIs or UC+ and *X* the time (months of evolution). For testing the regression lines for parallelism (i.e., equality of slopes) the *t* test was used [17].

## Results

### Clinical profile

Both groups of patients were similar in terms of age, time of evolution of UTIs, and the mean number of UTIs and UC+ for a period of 6 months (Table 1) before the initiation of the treatments. They were also similar in terms of sexual activity, menopause status, records of eutocic or dystocic childbirth, multiparous or nulliparous status, allergies, arterial hypertension, type 1 and 2 diabetes, smoking habit, obesity (body mass index >30), cystocele > grade 2, treatment for depression, breathing or stomach disorders, surgical background including hysterectomy, double oophorectomy, surgery for urinary incontinence (Marshall-Marchetti-Krantz colposuspension, tension-free vaginal tape, or transobturator vaginal tape), pelvic organ prolapse repair, or race (Table 2).

**Table 2:** Distribution of the clinical variables in women with repeated UTI

	Group A	Group B	*P* ^a^
Age (range)	47.7 (45.0-50.4)	48.1 (45.1-51.0)	0.8525
Regular sexual activity	130	122	0.2716
Menopause	64	75	0.2594
Eutocic childbirth	90	87	0.7358
Dystocic childbirth	28	33	0.5694
Multiparous	61	72	0.2566
Nulliparous	41	40	0.8982
Drug allergy	32	29	0.6716
Arterial hypertension	41	52	0.2181
Diabetes mellitus	24	29	0.5478
Smoking habit	43	50	0.4602
Obesity	30	45	0.0642
Cystocele > grade 2	0	0	1.0000
Antidepressant/anxiolytic drugs	58	61	0.8171
Breathing disorders	22	29	0.3596
Stomach disorders	39	45	0.5254
Hysterectomy	50	56	0.5527
Double oophorectomy	42	49	0.4573
Surgical correction of urinary incontinence	38	31	0.3439
Surgical correction of cystocele	18	15	0.5868
Caucasian race	150	149	0.8180

^a^Age assessed with Student’s *t* test and other variables with Fisher’s exact test

### Safety

Regarding Uromune®, none of the patients reported any side effects, either local at the site of administration or systemic.

### Urinary tract infections

**Fig. 1:**
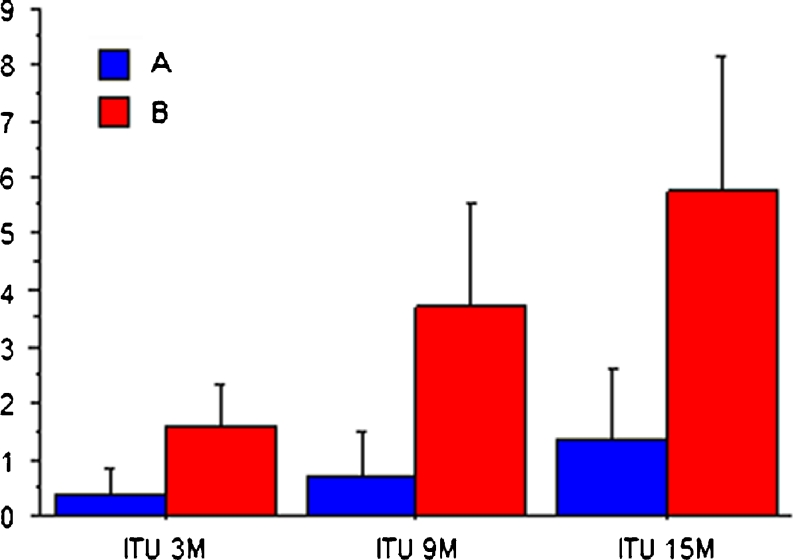
Average of UTIs at the 3-, 9-, and 15-month evaluation time points. *Error bars* are ± 1 standard deviation

The group treated with Uromune® experienced a highly significant reduction in the number of infections compared to group B. In the first 3 months the mean number of infections was 0.36 and 1.60 (*P* < 0.0001), respectively. A highly significant reduction was also observed after 9 and 15 months (*P* < 0.0001) (Table 3, Fig. 1). The improvement of the patients in group A compared to group B was 75 % in the first 3 months and 86 and 77 % at 9 and 15 months, respectively. The statistical analysis of the evolution of the number of UTIs in both groups was significant (ANOVA, *P* < 0.001). The numbers of patients who did not suffer any UTI at 3, 9, and 15 months were 101, 90, and 55 in group A, whereas in group B there were 9, 4, and 0 (*P* < 0.0001) (Table 4).

**Table 3:** Mean number of UTIs and UC+ (with 95 % CI) between the different evaluation time points in the clinical history of the patients

	Group A	Group B	*P*
UTIs			
0–3 months	0.36 (0.29–0.44)	1.60 (1.49–1.71)	<0.0001
0–9 months	0.72 (0.60–0.85)	3.71 (3.43–3.99)	<0.0001
0–15 months	1.35 (1.15–1.54)	5.75 (5.37–6.13)	<0.0001
3–9 months	0.36 (0.24–0.47)	2.11 (1.89–2.34)	<0.0001
3–15 months	0.98 (0.79–1.17)	4.15 (3.83–4.47)	<0.0001
9–15 months	0.62 (0.48–0.77)	2.04 (1.85–2.23)	<0.0001
UC+			
0–3 months	0.50 (0.42–0.57)	3.18 (0.08–6.28)	<0.0001
0–9 months	1.06 (0.91–1.22)	9.95 (0.26–19.64)	<0.0001
0–15 months	1.34 (1.15–1.53)	15.18 (0.39–29.97)	<0.0001
3–9 months	0.57 (0.43–0.71)	3.41 (3.16–3.65)	<0.0001
3–15 months	0.84 (0.67–1.02)	6.04 (5.71–6.36)	<0.0001
9–15 months	0.77 (0.64–0.91)	2.63 (2.43–2.83)	<0.0001

**Table 4:** Number of patients free of UTIs and UC+ between the different evaluation time points in the clinical history of the patients

	Group A	Group B	*P*
UTIs			
0–3 months	101	9	<0.0001
0–9 months	77	4	<0.0001
0–15 months	55	0	<0.0001
3–9 months	126	32	<0.0001
3–15 months	90	6	<0.0001
9–15 months	108	23	<0.0001
UC+			
0–3 months	80	5	<0.0001
0–9 months	56	0	<0.0001
0–15 months	49	0	<0.0001
3–9 months	111	3	<0.0001
3–15 months	95	1	<0.0001
9–15 months	70	6	<0.0001

### Urocultures

**Fig. 2:**
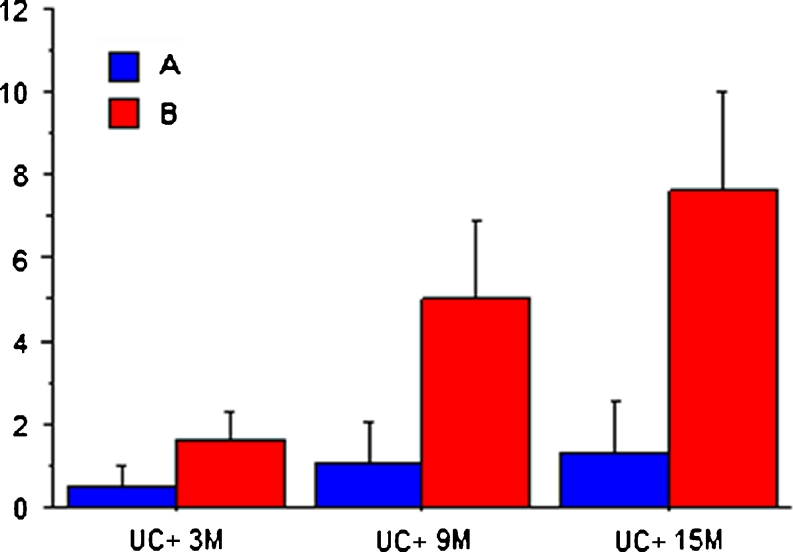
Average of UC+ at the 3-, 9-, and 15-month evaluation time points. *Error bars* are ± 1 standard deviation

In the 3 months previous to the initiation of treatment, the total number of UC+ in both groups was 902 (422 in group A and 480 in group B). Once the prophylactic treatment was initiated, group A patients had significantly less UC+ than group B. The mean of UC+ at 3, 9, and 15 months was 0.50, 1.06, and 1.34 in group A versus 1.60, 5.01, and 7.64 in group B. The differences between both groups at each evaluation time point were highly significant (*P* < 0.0001) (Table 3, Fig. 2). The statistical analysis of the evolution of the number of UC+ (ANOVA) was significant (*P* < 0.001) in both groups. The numbers of patients who did not have any UC+ at 3, 9, and 15 months were 80, 56, and 49 in group A, whereas in group B there were 5, 0, and 0 (*P* < 0.0001) (Table 4). The bacteria isolated in the urocultures are shown in Table 5.

**Table 5:** Microorganisms involved in UC+ in the 3 months previous to the initiation of the prophylactic treatments and at 3, 9, and 15 months of evaluation

3 months before the treatment							3 months				9 months				15 months			
	A	%	B	%	A + B	%	A	%	B	%	A	%	B	%	A	%	B	%
*Escherichia coli*	302	71.6	426	88.8	728	80.7	53	70.7	201	79.4	112	70.9	616	79.6	144	73.1	902	76.1
*Klebsiella pneumoniae*	14	3.3	11	2.3	25	2.8	1	1.3	20	7.9	6	3.8	50	6.5	7	3.6	62	5.2
*Klebsiella oxytoca*	12	2.8	14	2.9	26	2.9	4	5.3	7	2.8	5	3.2	27	3.5	5	2.5	49	4.1
*Proteus mirabilis*	11	2.6	2	0.4	13	1.4	3	4.0	5	2.0	7	4.4	16	2.1	11	5.6	33	2.8
*Streptococcus agalactiae*	18	4.3	4	0.8	22	2.4	3	4.0	1	0.4	5	3.2	4	0.5	5	2.5	9	0.8
*Staphylococcus epidermidis*	10	2.4	5	1.0	15	1.7	3	4.0	3	1.2	6	3.8	13	1.7	6	3.0	28	2.4
*Staphylococcus saprophyticus*	12	2.8	7	1.5	19	2.1	0	0.0	7	2.8	2	1.3	9	1.2	2	1.0	17	1.4
*Enterobacter cloacae*	6	1.4	3	0.6	9	1.0	1	1.3	3	1.2	2	1.3	10	1.3	1	0.5	29	2.4
*Enterobacter aerogenes*	9	2.1	0	0.0	9	1.0	1	1.3	0	0.0	1	0.6	0	0.0	2	1.0	0	0.0
*Enterococcus faecalis*	21	5.0	5	1.0	26	2.9	4	5.3	4	1.6	9	5.7	13	1.7	9	4.6	30	2.5
*Citrobacter koseri*	2	0.5	3	0.6	5	0.6	2	2.7	2	0.8	1	0.6	16	2.1	3	1.5	26	2.2
*Raoultella planticola*	2	0.5	0	0.0	2	0.2	0	0.0	0	0.0	1	0.6	0	0.0	1	0.5	0	0.0
*Pseudomonas aeruginosa*	3	0.7	0	0.0	3	0.3	0	0.0	0	0.0	1	0.6	0	0.0	1	0.5	0	0.0

### Regression analysis

**Fig. 3:**
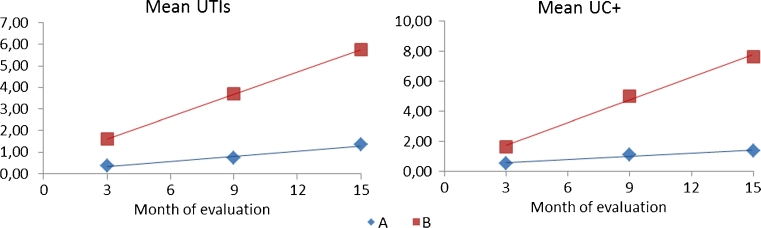
Regression lines of the evolution in the number of UTIs and UC+ along the time of evaluation

Table 6 and Fig. 3 show the parameters of the regression analysis between the mean number or UTIs or UC+ and the time of observation (slope, intercept, correlation coefficient, standard error of the slope, and the *t* test for parallelism). The regression lines were not parallel, since the slope of evolution of UTIs in group B was 4.23 times bigger than the slope of group A. In the case of UC+, the slope was 7.16 times bigger.

**Table 6:** Parameters of the regression lines corresponding to the evolution in the number of UTIs and UC+ along the time of evaluation

		a	b	*r* ^2^	Sb	*t*	*P*
UTI	A	0.075	0.082	0.999	0.012	6.789	0.021
	B	0.575	0.346	0.976	0.037		
UC+	A	0.334	0.070	0.994	0.014	12.345	0.007
	B	0.437	1.000	0.962	0.074

## Discussion

In this retrospective observational study we collected the data from the clinical histories of the clinical evolution of 319 women affected by RUTIs, comparing the clinical impact of the prophylactic treatment with a bacterial vaccine (Uromune®) and the currently accepted antibiotic therapy. We established the composition of this named patient bacterial preparation, for the prophylactic treatment of our patients, on the basis of the most common bacteria causing urinary infections in Spain [14]. Group A patients were treated with Uromune® for a period of 3 months without reporting any side effects and having an improvement of 75 % in the number of UTIs when compared to the number of UTIs of group B in the same period of time, although this group was under prophylactic antibiotic treatment for a period of 6 months. The benefit of Uromune® was maintained after an observation period of 9 and 15 months (86 and 77 % of improvement, respectively).

Bacterial preparations have been used to prevent RUTIs. Lettgen [18] reported the use of an oral bacterial lysate of 10^9^ *E. coli* compared with the use of nitrofurantoin as a prophylaxis of RUTIs in girls, showing that the efficacy of the long-term administration of this bacterial lysate was comparable to that of nitrofurantoin. Bauer et al. [19] reported in 2002 a meta-analysis performed on five studies of an oral bacterial lysate of 10^9^ *E. coli* compared to placebo in double-blind studies in patients with UTI (601 women), showing superiority of this treatment over placebo, with a confidence interval of 0.64-0.72. The drug was well tolerated and patient compliance was excellent in all studies. The same authors reported in 2005 [20] a double-blind, placebo-controlled study in 454 women using the same product. Patients were treated with 1 capsule (active or placebo) per day for 90 days, 3 months without treatment, then the first 10 days in months 7, 8, and 9 and were followed up during 12 months. The authors reported a 34 % reduction of UTIs in patients treated with the bacterial lysate when compared to placebo. In our study, patients were treated for a period of 3 months and showed a reduction of 75, 86, and 77 % at 3, 9, and 15 months when compared with conventional antibiotic prophylaxis, not to placebo. This high difference in the clinical benefit could be explained by the form in which the antigen is delivered (lysate or whole inactivated bacteria) and the route of administration (orally swallowed or sublingual).

Purified components from bacteria selectively activate Toll-like receptors (TLR), leading to shared and unique responses in innate immune cells, whereas whole non-lysate bacteria contain agonists for multiple TLR and induce a common macrophage activation program [21] eliciting a more potent and robust response [22], because the innate immune response to whole bacteria is a consequence of the cumulative activation of TLR [23].

These products act through the mucosal immune system. This local system contributes almost 80 % of all immunocytes. These cells are accumulated in, or in transit between, various mucosa-associated lymphoid tissues (MALT), which together form the largest mammalian lymphoid organ system [24] and are considered as the “common mucosal immune system” whereby immunocytes activated at one site disseminate immunity to remote mucosal tissues, although there is a significant degree of compartmentalization linking specific mucosal inductive sites with particular effector sites [24]. Oral immunization (swallowed) may induce substantial immune responses in the small intestine (strongest in the proximal segment), ascending colon, and mammary and salivary glands, but it is relatively inefficient at evoking response in the distal segments of the large intestines, tonsils, or female genital tract mucosa [25–27]. However, sublingual and nasal mucosa can serve as an inductive site for generating a broad spectrum of mucosal and systemic immune responses, including also the respiratory and genitourinary tracts [24, 28] with a high degree of efficacy and persistence of the immune response [29]. Sublingual administration of immunogens such as cholera toxin and ovalbumin induces systemic humoral dose-dependent immune responses [28], mucosal antibody responses [28], and an immune-stimulating effect on CD4+ T helper cell responses to bacteria [30].

The number of UTIs increases after the 3 months of treatment with Uromune®, being more pronounced from the 9th month of observation. This observation highlights the need for a more prolonged treatment or for starting a new period of 3-month treatment. In a pilot trial using a similar immunostimulant, with a different bacterial composition because it focused on preventing recurrent respiratory tract infections, the treatment period was of 6 months’ duration and had a clinical effect for a minimum period of 12 months [30].

An important finding, not described previously, is the trend of developing UTIs and the presence of UC+ by means of the regression analysis. The corresponding slopes in group A are much flatter than those of group B, indicating that the trend to develop UTIs and to have UC+ is much more meaningful in group B and showing that for one UTI in group A there are more than four UTIs in group B and for one UC+ in group A there are more than seven in group B.

The decrease in the number of UTIs implies an important decrease in antibiotic consumption. This approach is in line with the recommendations of health authorities, because the use of this immunostimulant agrees with the need for new treatment alternatives, non-antimicrobial treatments (vaccines) against bacterial diseases and diseases that may precipitate secondary bacterial diseases [31–35].

This study demonstrates the effectiveness of a bacterial preparation administered through the sublingual route. We acknowledge that because this study is retrospective, it does not provide deeper and more accurate outcomes as could be obtained in a prospective explanatory double-blind placebo-controlled trial conducted under clinical experimental conditions. However, the data collected from clinical histories of patients provide clinically valuable information of the patients treated under “real-life” conditions.

However, we believe that further prospective double-blind, placebo-controlled, randomized clinical trials are needed to establish more accurately the clinical impact (severity of episodes of UTI, quality of life, associated economic costs) of these bacterial immune stimulants in patients with RUTIs, although the Note of Clarification on Paragraph 29 added by the World Medical Association General Assembly to the Declaration of Helsinki [36] states that care must be taken in using a placebo-controlled trial and that this methodology should only be used in the absence of an existing proven therapy.

Considering the clinical impact due to the high prevalence and the high cumulative cost of UTIs, together with the increasing resistance to antibiotics, the results obtained in this study favor the use of bacterial immunostimulation, which could be an effective strategy to reduce frequency, duration, severity, and costs of RUTIs in adults and children.
